# The critical role of gut microbiota in obesity

**DOI:** 10.3389/fendo.2022.1025706

**Published:** 2022-10-20

**Authors:** Zilu Cheng, Li Zhang, Ling Yang, Huikuan Chu

**Affiliations:** Division of Gastroenterology, Union Hospital, Tongji Medical College, Huazhong University of Science and Technology, Wuhan, China

**Keywords:** gut microbiota, obesity, energy homeostasis, bile acids, SCFAs

## Abstract

Obesity is a global epidemic characterized by energy disequilibrium, metabolic disorder, fat mass development, and chronic low-grade inflammation, which significantly affects the health state of individuals of all ages and strains the socioeconomic system. The prevalence of obesity is rising at alarming rates and its etiology involves complicated interplay of diet, genetic, and environmental factors. The gut microbiota, as an important constituent of environmental factors, has been confirmed to correlate with the onset and progression of obesity. However, the specific relationship between obesity and the gut microbiota, and its associated mechanisms, have not been fully elucidated. In this review, we have summarized that the microbial diversity was significantly decreased and the Firmicutes/Bacteroidetes ratio was significantly increased in obesity. The altered gut microbiota and associated metabolites contributed to the progression of the disease by disrupting energy homeostasis, promoting lipid synthesis and storage, modulating central appetite and feeding behavior, as well as triggering chronic inflammation, and that the intentional manipulation of gut microbiota held promise as novel therapies for obesity, including probiotics, prebiotics, and fecal microbiota transplantation.

## 1 Introduction

It is widely acknowledged that obesity is a chronic metabolic disease mainly induced by the disequilibrium of energy intake and energy expenditure, which results in excess fat accumulation, metabolic disorders, as well as chronic low-grade inflammation. The prevalence of obesity is rising at alarming rates (1). According to a comprehensive study, overweight and obese individuals account for about one-third and 10% of the world’s population, respectively (2). Besides, 1.12 billion people in the globe are predicted to suffer from obesity by 2030 (1), which poses a great threat to human health and an enormous burden to the social economy. Moreover, obesity has been considered to correlate with higher risks of other diseases, such as cardiovascular and respiratory problems, diabetes, and even cancer (2, 3). The etiology of obesity is multifactorial and not yet fully elucidated; some factors include sedentary lifestyle, unhealthy eating habits, genetic predisposition, and environmental factors (4, 5). Evidence is mounting that the gut microbiota, as a pivotal environmental factor, contributes to the occurrence and progression of obesity significantly, which has been confirmed to play significant roles in other metabolic disorders, such as non-alcoholic fatty liver disease and diabetes (6–9).

The gut microbiota refers to a complicated ecosystem colonized in the human gut tract, which embodies large amounts of microorganisms, including bacteria, fungi, virus, archaea, protists and so on. The total weight of the gut microbiota is about 1-2 kg, and the number of genes it contains is more than 100 times than that of the human body in which it resides (10). In a healthy status, the gut microbiota coexists with the host harmoniously and participates in the regulation of multiple physiological functions of the host, including digesting and absorbing essential nutrients, conferring protection against detrimental microbes, and maintaining immune homeostasis (11). Herein, gut dysbiosis is unfavorable for the host, and putatively results in a variety of diseases, including obesity, which alignes with previous studies (6–9). The altered gut microbiota putatively participates in the pathogenesis of obesity *via* multiple mechanisms, including energy homeostasis disruption, lipid synthesis and storage, central appetite and feeding behavior regulation, as well as chronic low-grade inflammation.

Currently, there exist various effective interventions for obese treatment, such as healthy lifestyles, weight-reducing drugs, and bariatric surgery. Nevertheless, great efforts are still warranted to seek novel validated therapeutic methods, owing to the difficulty of sustaining long-term diet control, moderate exercise, and the undesirable effects of drugs and surgery (12). On the other hand, gut microbiota could be a promising target for obese treatment as it is a critical contributor to the progression of obesity. Therefore, more attention should be paid to elucidating the relationship between obesity and the gut microbiota, unraveling the underlying mechanisms that the gut microbiota induces obesity, as well as exploring the safety and efficacy of potential therapies based on gut microbiota restoration for obesity treatment.

## 2 The relationship between obesity and gut microbiota

### 2.1 Gut bacteria

The gut bacteria play critical roles in the onset and progression of obesity. Following the same high-fat diet (HFD) feeding, the wild-type mice developed obesity, whereas the germ-free mice did not, indicating that germ-free mice were able to resist HFD-induced obesity (6). *Enterobacter cloacae*, which was intimately related to obesity, brought reduced adiponectin levels, elevated lipopolysaccharide binding protein (LBP) concentration, glucose tolerance disruption, and weight gain upon introduction into the germ-free mice (7). Moreover, the germ-free mice developed obesity when transplanted with fecal microbiota from obese mice (8, 9). Conversely, the symptom of metabolic syndrome in obese mice was markedly ameliorated after they received fecal microbiota from lean mice (13). The above noted evidence supported the notion that gut bacteria are intimately associated with obesity.

An accumulating body of evidence has suggested that the composition and biodiversity of gut bacteria in obese groups significantly differed from those in healthy groups (**Table 1**) (14–20). Compared to the controls, the diversity of gut bacteria has decreased significantly in obese subjects (20). The taxonomic analysis of fecal bacteria from obese individuals and lean individuals showed that at the phylum level, the abundance of Firmicutes and the Firmicutes/Bacteroidetes ratio have increased significantly in obese subjects while the abundance of Bacteroidetes has decreased significantly in such groups compared to lean subjects (19). These findings were consistent with the results reported by previous animal studies that analyzed the fecal microbiome of lean mice and obese mice induced by HFD (21). The reduction of Bacteroidetes was thought to correlate with fat loss, whereas the increment of Firmicutes was positively related to digestible energy intake and fat storage (18). However, in several recent studies, some researchers have found that no significant difference existed in obese and lean individuals concerning Firmicutes/Bacteroidetes ratio and the abundance of Bacteroidetes (16, 18). The controversial results warrant further investigation. At the genus level, the abundance of *Lactobacillus reuter*, *Fusobacteria Alistipes, Anaerococcus, Corpococcus, Fusobacterium*, *Parvimonas, Bifiobacterium*, *Clostridium leptum*, *Lactobacillus/Leuconostoc/Predicoccus, Veillonellaceae, Paraprevotellaceae*, *Roseburia* sp., and *Eubacterium* sp. were enriched in obese subjects compared to lean subjects (14, 16–18, 20) In contrast, the abundance of *Akkermansia, Lactobacillus plantarum, Clostridium leptum, Clostridium coccoides, Bifidobacterium longum, Bifidoacterium animalis, Lactobacillus Plantarum, Lactobacillus paracasei, Methanobrevibacter smithii, Bacteroides, Desulfovibrio, Faecalibacterium, Lachnoanaerobaculum, Olsenella*, *Prevotella, Eggerthella, Adlercreutzia*, *Bacteroides rodentium, B. intestinalis*, and *B. eggerthii* were retracted in such subjects (14–18, 20). The afore-mentioned findings also indicated the specificity of obesity-related bacteria species, and more precisely, bacteria from the identical genus exhibited contrary functions in obesity, which the complicated metabolic mechanisms of obesity could partly explain.

**Table 1:** Alterations of gut bacteria associated with obesity in humans.

Study	Participants	Comparison	Change of gut microbiota		Method
			Increased	Decreased
Million et al. (14)	Obese (n=68) Lean (n=47)	Obese vs Lean	*Lactobacillus reuter*	*Methanobrevibacter smithii*, *Lactobacillus paracasei, Lactobacillus* *Plantarum, Bifidoacterium animalis*	qPCR
Teixeira et al. (15)	Obese (n=17) Lean (n=15)	Obese vs Lean		*Bifidobacterium, Bifidobacterium* *longum*, *Clostridium coccoides*, *Clostridium* *leptum*, *Lactobacillus* *plantarum, Akkermansia*	qPCR
Andoh et al. (16)	Obese (n=10) Lean (n=10)	Obese vs Lean	Firmicutes, *Fusobacteria* *Alistipes, Anaerococcus, Corpococcus, Fusobacterium Parvimonas*	*Bacteroides, Desulfovibrio, Faecalibacterium, Lachnoanaerobaculum* *Olsenella*	16S rRNA sequencing
Selma et al. (17)	OW and Obese (n=49) Lean (n=20)	OW and obese vs Lean	Firmicutes, *Clostridium* *leptum, Lactobacillus/* *Leuconostoc/* *Predicoccus, Bifidobacterium*	*Prevotella*	qPCR
Yun et al. (20)	OW (n=326) Obese (n=419) NW (n=529)	OW and obese vs NW	*Veillonellaceae*, *Paraprevotellaceae*,	*Akkermansia*, *Eggerthella, Adlercreutzia*	16S rRNA sequencing
Kolida et al. (19)	Obese (n=11) UW (n=7) NW (n=17)	Obese vs UW and NW	Firmicutes	Bacteroidetes	16S rRNA sequencing
Murga-Garrido et al. (18)	OW and obese (n=20) NW (n=26)	OW and obese vs NW	*Eubacterium sp.* *Roseburia sp.*	*Bacteroides rodentium*, *B. intestinalis*, *B. eggerthii*, *Methanobrevibacter smithii*	genomic pool sequencing

Comparison of condition A vs condition B: “Increased” signifies an increase in condition A relative to condition B. “Decreased” signifies a decrease in condition A relative to condition B.

OW, overweight; NW, normal weight; UW, underweight.

In addition, some specific species had an intimate association with the degree of obesity and the levels of associated metabolic indicators. The *Lactobacillus* genera were negatively related to a body mass index, a proxy for adiposity, and positively correlated with leptin independent of calorie intake (22). In several studies, the *Christensenellaceae* was negatively related to total cholesterol, serum triglyceride, low density lipoprotein, and apolipoprotein B, while it had a positive correlation with high density lipoprotein (23).

### 2.2 Gut non-bacterial communities

Besides bacteria, gut archaea, fungi and virus have contributed to the pathogenesis of obesity. Zhang et al (24) found that compared to post-gastric-bypass or normal weight subjects, the abundance of H2-utilizing methanogenic Archaea has increased significantly in obese subjects. When coexisting with H2-producing bacteria in the human gut tract, the H2-utilizing methanogenic Archaea had the capacity to facilitate the interspecies H2 transfer between bacteria and archaea, which was considered as one of the crucial approaches to enhance energy uptake in obese subjects (24). Moreover, the increment of H2-utilizing methanogenic Archaea was also positively related to short-chain fatty acids (SCFAs) production by facilitating fermentation, which played significant roles in the progression of obesity (24).

Besides, the abundance of the fungal species Saccharomyces cerevisiae has increased significantly in the obese mice induced by HFD, compared to lean mice (25). Conversely, the Saccharomyces species abundance in obese individuals has decreased significantly compared to the controls in a recent report (26). Whether this alteration in fungal species will exert influence on the development of obesity and the specific related mechanisms remains largely unknown.

It has been observed that the amount of weight gain of obese mice was reduced after they were transplanted with caecal viral communities from lean subjects, which supported the intimate linkage between gut virus and obesity (27). The amounts of viral RNA and DNA in fecal samples from obese mice have increased significantly compared to those in normal mice, which indicated the significant increment of RNA and DNA viral communities in obese subjects (28). Moreover, fecal viral contents have been confirmed to have a positive correlation with obese-enriched bacteria, such as Firmicutes, whereas fecal viral population was negatively related to lean-enriched bacteria, including Bacteroidetes and *Bifidobacteria (* 29). It seems reasonable to speculate that gut viral communities putatively take part in the development of obesity by interacting with gut obese-related bacteria, especially bacteriophages. Furthermore, the increment of fecal viral communities was putatively conducive to the release of viral proteins (28), which had the capacity to interact with host cells and take part in the regulation of biological processes, such as host metabolism and inflammation responses (30), thereby contributing to the progression of obesity.

### 2.3 The differences of gut-microbiota patterns in obesity and type-2 diabetes mellitus

Obesity and type-2 diabetes mellitus (T2DM) are both emerging as global epidemics, and they encompass common underpinnings, including insulin resistance, dysglycemia as well as chronic low-grade inflammation. A plethora of researches have indicated that gut microbiota plays a critical role in the etiology of obesity and T2DM (31, 32). Not surprisingly, there are some similar alterations of the gut microbiota in obesity and T2DM owing to their common pathological characteristics. To be more specific, the abundance of Gram-negative bacteria was increased in both obesity and T2DM groups, which triggers chronic low-grade inflammation in these metabolic diseases (31). Moreover, the genera of *Akkermansia*, *Faecalibacterium*, and *Bacteroides* have a negative association with obesity and T2DM (32). However, the differences in gut microbiota between obesity and T2DM still exist. At the phylum level, the Firmicutes abundance in T2DM was markedly decreased while the Bacteroidetes abundance was markedly increased compared to the controls (33), which was completely contrary to the corresponding results in obesity. In addition, unlike obesity, the abundance of butyrate-producing bacteria in T2DM was significantly decreased, such as *Faecalibacterium prausnitzii, Roseburia intestinalis, Bacteroides intestinalis, Eubacterium eligens*, and *Eubacterium rectal*e *(* 34, 35).Moreover, in contrast to obesity, the conditioned pathogens were markedly enriched in T2DM patients, including *Escherichia coli, Clostridium symbiosum, Clostridium ramosum, Clostridium hathewayi, Clostridium clostridioforme, Eggerthella lenta*, and *Bacteroides caccae (* 34–36). The analysis and comparison of the gut microbiota in obesity and T2DM are conducive to more precise treatment and management of the two diseases.

To sum up, gut microbiota has an intimate association with obesity. Compared to the controls, the reduced bacterial diversity and increased Firmicutes/Bacteroidetes ratio were generally regarded as the basic characteristics of obese subjects despite several controversial results (19–21). Some specific bacterial species, such as *Lactobacillus (* 22) and *Christensenellaceae (* 23), were related to the grade of obesity and the level of associated metabolic indicators in obese individuals. Besides bacteria, gut archaea, fungi, and viruses have also contributed to the pathogenesis of obesity (24–28). However, studies concerning their specific roles in the occurrence and development of the disease are still in their infancy, awaiting further investigation and the emergence of new evidence. Furthermore, the gut microbiota composition in obesity is different from that in other metabolic diseases, such as T2DM (33–36), and great efforts are still warranted to identify the specific species of gut microbiota in obesity.

## 3 The mechanisms by which the gut microbiota influences obesity

### 3.1 Energy homeostasis disruption

#### 3.1.1 Digestible energy uptake

Evidence has been mounting that altered gut microbiota in obese subjects exhibited more potent energy uptake properties from ingested food compared to the controls, mainly by promoting the production of nutrient transporters and various primary fermentation enzymes (37, 38). To be more specific, the increment of *Clostridium ramosum* (Firmicutes phylum), was able to increase the efficiency of digestible energy uptake *via* higher expression of Glut2 (a glucose transporter) and CD36 (a fatty acid translocase) (37). The increased Firmicutes abundance and Firmicutes/Bacteroidetes ratio in obese subjects correlated with the digestion of some indigestible polysaccharides, the subsequent production of monosaccharides and SCFAs, especially acetate and butyrate, and the energy extraction from substances that would alternatively have been diminished by the faeces. These processes were predominantly induced by elevated levels of α-amylases and amylomaltases in obese-enriched gut bacteria (38). The host can absorb the produced monosaccharides and SCFAs in the gut tract. And it is estimated that SCFAs can provide humans with 5-15% of the total calories required and provide colonic epithelial cells with 60-70% of the calories required (39).

In addition, the interspecies H2 transfer between bacteria and archaea also greatly increased digestible energy uptake in obese subjects (24). The parallel increment of H2-utilizing methanogenic Archaea and H2-producing bacteria in obese individuals’ gut tract was conducive to the conversion of polysaccharides to SCFAs, mainly through relieving thermodynamic limitations, as H2-utilizing methanogenic Archaea had the capacity to dislodge fermentation intermediates, such as formate or H2 (24).

#### 3.1.2 Energy expenditure

The changed gut microbiota in obese subjects leads to the alterations of luminal contents metabolism, such as bile acids and SCFAs, which participate in the modulation of energy expenditure. The activated Takeda G protein-coupled receptor5 (TGR5) by the bile acid in brown adipose tissue (BAT) induces the expression of PPARγ coactivator-1α (40) and iodothyronine-deiodinase type 2. The former is a significant regulator of mitochondrial biogenesis, and the latter is conducive to the transition from inactive thyroxine to 3,5,3’-triiodothyronine, which further enhances the uncoupling of mitochondrial function and increasing thermogenesis *via* the activation of the thyroid hormone receptor, ultimately contributing to energy expenditure. Moreover, in the intestine, the activated Farnesoid X Receptor (FXR) was able to enhance fibroblast growth factor (FGF) 15/19 secretion, which has the capacity to increase the production of TGR5 ligand and induce the alterations of bile acid pool composition, thereby resulting in white adipose tissue browning, BAT activation, and more thermogenesis (41). These alterations are all favorable for energy expenditure. Therefore, the reduction of bile acids induced by gut dysbiosis in obesity, such as the decreased *Bacteroides* and *Lactobacillus (* 42), undermines energy expenditure mainly by the inactivation of afore-mentioned bile acids-mediated signaling pathways, thus exacerbating the disease’s progression.

SCFAs are converted from indigestible dietary fiber *via* the fermentation process by the gut microbiota. They are predominantly comprised of acetic acids, propionic acids, and butyric acids. The concentration of SCFAs has increased in obese subjects mainly owing to their elevated level of Firmicutes and H2-utilizing methanogenic Archaea (24, 43). SCFAs also participate in the regulation of energy expenditure, but its function seems controversial. On the one hand, in the presence of SCFAs, the secretion of fasting-induced adipose factor (FIAF) from the gut is suppressed (44, 45). FIAF has the capacity to increase the activity of AMP-activated kinase that is extensively distributed in the skeletal muscle, liver as well as colon, and further promote the catabolic process such as β-oxidation (46), thus conferring protection against obesity. On the other hand, butyrate, as the most significant component of SCFAs, has the capacity to phosphorylate AMP-activated kinase distributed in muscle and liver, as well as promote mitochondrial uncoupled protein 1 and PPAR-γ coactivator 1α expression in BAT, thus enhancing thermogenesis and fatty acid oxidation (47). Taken together, SCFAs seem a double-edged sword in energy expenditure modulation, which warrants more in-depth investigation.

**Figure 1:**
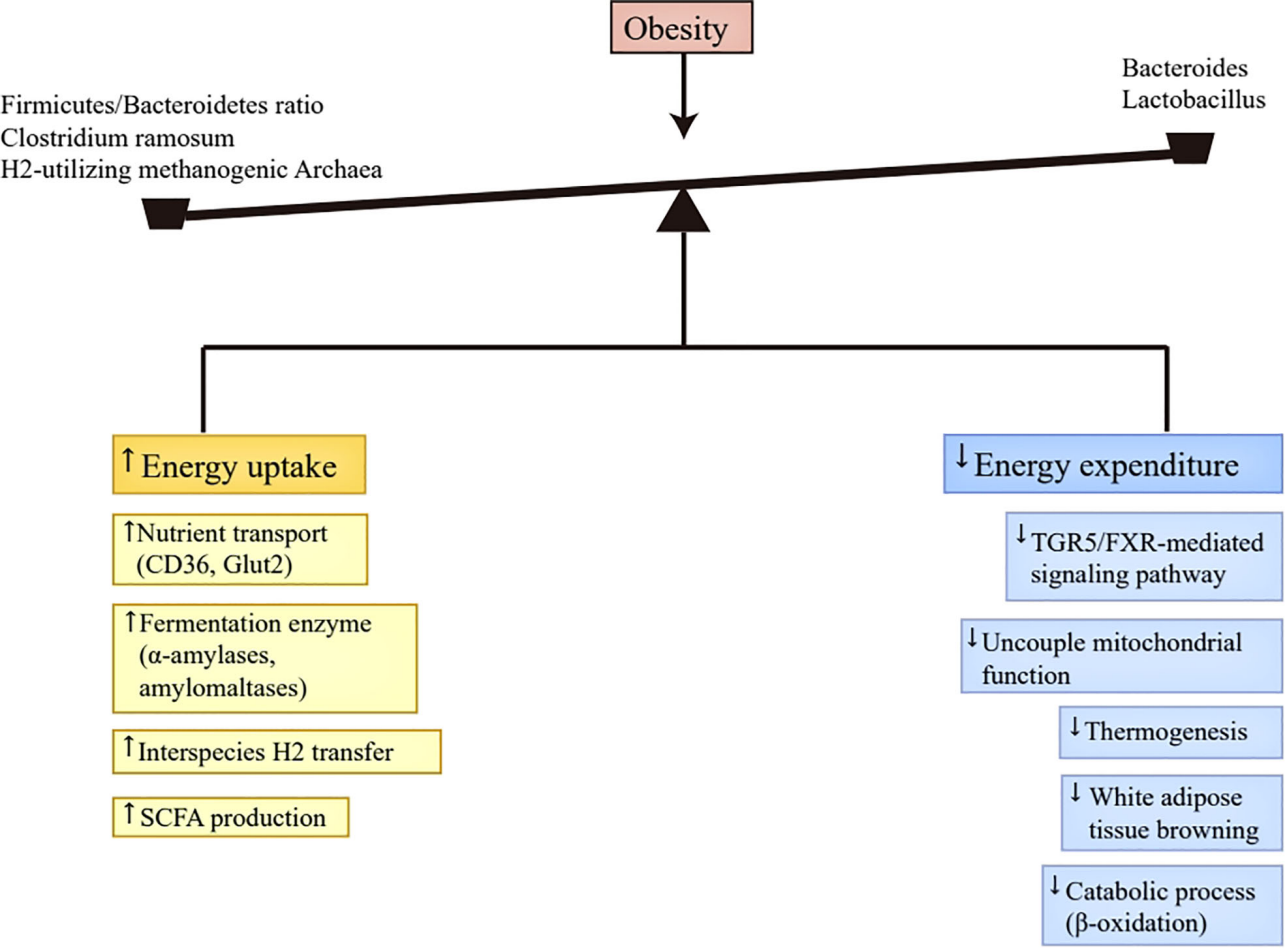
Energy homeostasis disruption. Abundant nutrient transporters (CD36, Glut2) and various primary fermentation enzymes (α-amylases and amylomaltases) caused by altered gut microbiota in obesity improve the efficiency of digestible energy uptake and promote monosaccharides and SCFAs production. Moreover, the production of SCFAs was also enhanced by the interspecies H2 transfer between bacteria and archaea. The produced monosaccharides and SCFAs in the gut tract can be absorbed by the host. The decrease of *Bacteroides* and *Lactobacillus* in obesity leads to the reduction of bile acids, which inactivates TGR5/FXR-mediated signaling pathway in brown adipose tissue, thus reducing uncouple mitochondrial function, thermogenesis, and white adipose tissue browning. Additionally, the presence of SCFAs suppresses FIAF secretion in the intestine, which then inhibits catabolic process, such as β-oxidation. Taken together, the altered gut microbiota in obesity results in more energy uptake and less energy expenditure, which contributes to the progression of obesity. SCFAs, short-chain fatty acids; TGR5, Takeda G protein-coupled receptor 5; FXR, Farnesoid X Receptor; FIAF, fasting-induced adipose factor.

To sum up, the altered gut microbiota contributed to obesity’s progression partly through increasing digestible energy intake and decreasing energy expenditure (**Figure 1**). Excess digestible energy intake was attributed to abundant nutrient transporters and various primary fermentation enzymes in obese-enriched gut bacteria (37, 38), and to the interspecies H2 transfer between bacteria and archaea (24), which increased the efficiency of digestible energy uptake and enhanced the production of SCFAs as energy substances. The changed gut microbiota also altered the luminal contents concentration by affecting their metabolism, including the decrease of bile acids (42) and the increase of SCFAs (43). The reduction of bile acids undermines energy expenditure *via* the inhibition of TGR5/FXR-mediated signaling pathways in adipose tissue (40, 41, 48). The increment of SCFAs can decelerate metabolic processes by repressing FIAF (44, 45).In addition, SCFAs also has the capacity to enhance fatty acid oxidation and thermogenesis through phosphorylating AMP-activated kinase and promoting mitochondrial uncoupled protein 1 and PPAR-γ coactivator 1α expression (47). Therefore, the function of SCFAs in regulating energy expenditure is controversial, and further investigation is warranted.

### 3.2 Lipid synthesis and storage

The altered gut microbiota in obese subjects exerts influence on lipid synthesis *via* multiple mechanisms. As mentioned above, the concentration of bile acids has decreased in obese subjects owing to the reduced *Bacteroides* and *Lactobacillus (* 42). Bile acids play significant roles in regulating lipid synthesis. The activated FXR by the bile acid in the liver restrains liver receptor homologue 1 expression in a small heterodimer partner-mediated way, and further inhibits sterol regulatory binding protein1c (SREBP1c) transactivation, which has an intimate association with the genes involved in lipogenesis, thus repressing hepatic *de novo* lipogenesis (49). Additionally, the release of FGF19 induced by FXR in the intestine can activate FGFR4 receptor on hepatocytes, and then inhibit SREBP1c directly by repressing peroxisome proliferator-activated receptor-γ coactivator 1β and inducing signal transducer and activator of transcription, and indirectly by enhancing SHP expression (50). Therefore, the decreased bile acids are conducive to hepatic *de novo* lipogenesis. Besides, the gut microbiota brings more digestible energy absorption and elevated serum glucose levels due to higher Glut2 expression AS a result, it promotes two basic transcriptional factor expressions, SREBP1 and carbohydrate response element binding protein (ChREBP), thus inducing hepatic lipid synthesis (51). Moreover, the increased SCFAs in obese individuals can rapidly be assimilated into host lipids and carbohydrates, especially acetates, which are identified as precursors for fatty acid or cholesterol synthesis (52). Butyrate is also favorable for lipid synthesis from ketone body or acetyl-CoA mainly by the activation of β-hydroxy-β-methylglutaryl-CoA pathway (53). Consistently, the amounts of lipolytic enzymes were decreased and the lipogenic genes expression was enhanced in the offspring after maternal butyrate supplementation (54). However, in another study, butyrate was found to downregulate the activity and expression of PPAR-γ, which facilitated the conversion of lipogenesis into lipid oxidation (55).

In addition, gut dysbiosis in obesity contributes greatly to lipid storage. The altered gut microbiota in obese subjects brings about higher lipopolysaccharide (LPS) concentration (20), which triggers a series of inflammation responses and induces metabolic endotoxemia (56, 57). In this context, the expression of proinflammatory cytokines in adipose tissues is significantly enhanced, including Interleukin (IL)-6 and tumor necrosis factor (TNF)-α (58), which can result in insulin resistance mainly *via* the inactivation of insulin receptor by phosphorylating the serine on it (59). Insulin resistance is favorable for excess lipid storage in adipose tissues and the liver. Additionally, metabolic endotoxemia had the capacity to increase adipocyte hyperplasia directly in CD14-dependent manner, and promote activin A production, which facilitated the proliferative process of adipocyte precursor cells (60). In a recent study, the gut microbiota induces fat mass storage by inhibiting the expression of *Gcg* and *Bdnf*, which encode body fat-suppressing neuropeptides, and by inducing leptin resistance mediated by Socs3 (61). Furthermore, in obese mice induced by HFD, some researchers have found that *L. paracasei* had the capacity to induce ANGPTL4 expression in the liver, which resulted in the inhibition of lipoprotein lipase (LPL) (62). LPL can assist the transport of triglycerides from the liver to systematic circulation, and then fat cells absorb them. Herein, it seems plausible to speculate that the reduction of *L. paracasei* in obese individuals contributes to lipid storage by releasing the inhibition of LPL.

**Figure 2:**
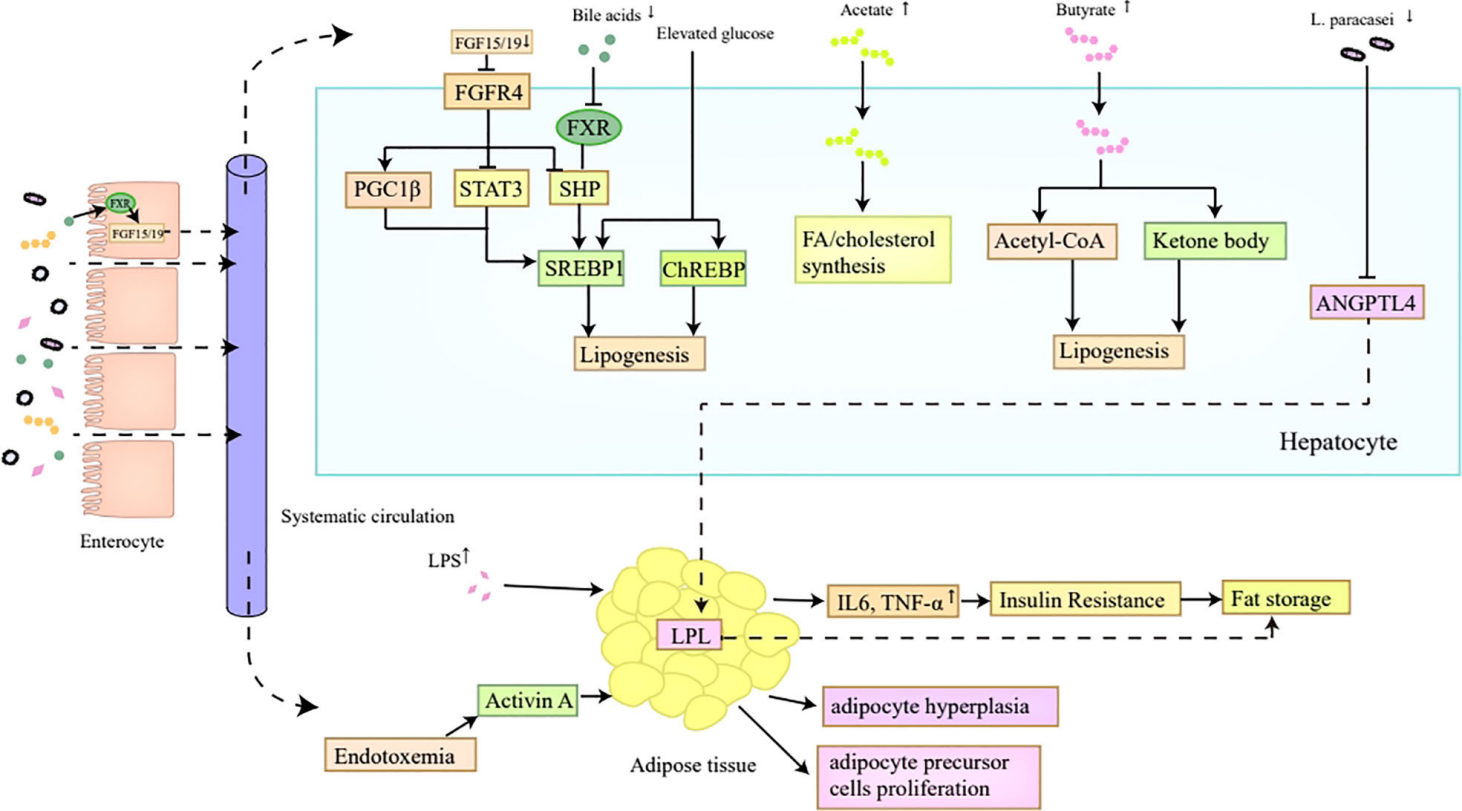
Lipid synthesis and storage. The reduction of bile acids in obesity inhibits FXR in hepatocytes, which ultimately induces the transactivation of SREBP1c mediated by SHP, thus contributing to hepatic *de novo* lipogenesis. Decreased bile acids are also unfavorable for FGF15/FGF19 secretion in the intestine, which then inactivates FGFR4 on hepatocytes and promotes SREBP1c transactivation indirectly by inhibiting SHP, and directly by inducing PGC1β and repressing STAT3, ultimately enhancing hepatic lipogenesis. Moreover, the SREBP1 and ChREBP expression can also be promoted by elevated glucose level which correlates with higher Glut2 expression induced by altered gut microbiota in obesity. In addition, the increased SCFAs in obesity, especially acetates and butyrates, can act as precursors for FA or cholesterol synthesis. The reduced *L. paracasei* in obese individuals putatively contributes to lipid storage by releasing the inhibition of LPL induced by ANGPTL4. Furthermore, elevated levels of proinflammatory cytokines and metabolic endotoxemia induced by LPS correlate with insulin resistance and the proliferation of adipocytes and adipocyte precursor cells, which all make contributions to lipid storage. FXR, Farnesoid X Receptor; SREBP1c, sterol regulatory binding protein1c; SHP, small heterodimer partner; FGF, fibroblast growth factor; PGC1β, peroxisome proliferator-activated receptor-γ coactivator 1β; STAT3, signal transducer and activator of transcription; ChREBP, carbohydrate response element binding protein; SCFAs, short-chain fatty acids; FA, fatty acids; LPL, lipoprotein lipase; LPS, lipopolysaccharide.

In summary, the gut microbiota promotes lipid synthesis and storage *via* multiple mechanisms (**Figure 2**). The reduction of bile acids contributes to hepatic *de novo* lipogenesis through promoting SREBP1c transactivation *via* the inactivation of FXR (49, 50). SCFAs can be regarded as precursors for fatty acid or cholesterol synthesis (52). Elevated levels of LPS-induced proinflammatory cytokines and metabolic endotoxemia correlate with insulin resistance and the proliferation of adipocytes and adipocyte precursor cells, resulting in excessive lipid storage (58–60). Besides, the gut microbiota is also favorable for lipid storage *via* the induction of leptin resistance and the inhibition of fat-suppressing neuropeptides (61). Moreover, the reduction of *L. paracasei* in obese individuals putatively contributes to lipid storage by releasing the inhibition of LPL according to an animal study (62).

### 3.3 Central appetite and feeding behavior

#### 3.3.1 The gut-brain axis

The gut can interact intimately with the central nervous system to transmit nutritional status information *via* multiple mechanisms, such as the gut endocrine system, the gut nervous system, and the vagus nerve (63).The complicated, continuous, and bidirectional crosstalk between the brain and gut is termed the gut-brain axis. In the wake of a more in-depth comprehension of the gut microbiota, many researchers have noticed that it played a crucial role in such a bidirectional communication, which is known as the microbiota-gut-brain-axis. Mounting studies have been accumulating that the microbiota-gut-axis was closely related to various gastrointestinal and nervous system diseases, including irritable bowel syndrome and Parkinson’s disease (64, 65). Given that the gut-brain axis participated in the modulation of central appetite and feeding behavior (66–71), the gut dysbiosis in obese subjects might exert influence on food intake, thus contributing to obesity’s progression.

#### 3.3.2 The role of gut microbiota in regulating central appetite and feeding behavior

The gut microbiota takes part in central appetite and feeding behavior modulation by influencing the production of bacterial metabolites, intestinal hormones as well as neurotransmitters. *Bifidobacterium* and *Lactobacillus* can produce lactate, which serves as a substrate for neuron cells, thus prolonging postprandial satiety (72). Acetates and butyrates are produced by bacterial fermentation from indigestible dietary fiber. Acetate is able to activate the citric acid cycle in the hypothalamus and further shift the expression profile of neuropeptides which regulate satiety (73). Butyrate can affect host appetite and feeding behavior by activating the vagus nerve and hypothalamus, as butyrate has the capacity to cross blood-brain barrier (74). The gut hormones, such as peptide YY (PYY) and glucogen-like peptide 1 (GLP-1), are secreted by enteroendocrine cells extensively distributed throughout the gut epithelium (75, 76). Bile acids, SCFAs, and indoles are intimately associated with the secretion of these gut hormones from enteroendocrine cells (77, 78). GLP-1 and PYY belong to potent anorexigenic hormones, which can impact host appetite and feeding behavior by binding to their receptors locally distributed in enteric neurons, vagal afferents, hypothalamus and brain stem (79, 80). The gut microbiota also leads to neurotransmitters production, including γ-aminobutyric acid (GABA) and serotonin (66), which correlate with central appetite control (67, 68). More specifically, GABA, as the predominant inhibitory neurotransmitters of the host nervous system, has the capacity to stimulate feeding (68). Serotonin can mainly suppress appetite by regulating melanocortin neurons, which contribute to maintaining of body weight homeostasis (81, 82).

In addition, the gut microbiota also affects central appetite and feeding behavior *via* the regulation of mood and reward pathways. The gut microbiota alters mood by producing microbial metabolites, activating immune responses, and stimulating the vagus nerve (69). When the psychological pressure of the host increases, the hedonic signaling pathways will be activated, thus increasing the host’s intake of high-calorie food (70). Herein, it seems reasonable to speculate that the gut microbiota affects mood, which then affects the brain circuits associated with feeding behavior. In a human imaging study, the increasing amount of propionate produced by bacteria fermentation, is related to the reduction of anticipatory reward response to high-calorie food mainly by striatal pathways (71), thus affecting feeding behavior.

In summary, the gut microbiota modulates central appetite and feeding behavior *via* a variety of mechanisms. On the one hand, the gut microbiota affects the production of bacterial metabolites, intestinal hormones, and neurotransmitters, which act as significant messengers in the interaction of the gut and brain, and further regulate host appetite and feeding behavior (66–68). On the other hand, the gut microbiota also takes part in the regulation of mood (69, 70) and reward pathways (71), which putatively affect the brain circuits associated with feeding behavior. It may be safe to draw a conclusion that the gut microbiota is closely related to the pathogenesis of obesity owing to its crucial function in regulating central appetite and feeding behavior.

### 3.4 Chronic inflammation

Chronic low-grade inflammation is generally regarded as one of the fundamental characteristics of obesity, which is mainly triggered by LPS (56, 57). LPS belongs to one kind of endotoxin, and is released by Gram-negative bacteria. The overgrowth of Gram-negative bacteria in obese individuals, such as *Veillonella*, can lead to a higher dose LPS in the intestine (20). The increment of LPS can destruct the gut barrier *via* the activation of the TLR4/MyD88/IRAK4 signaling pathway in the intestinal epithelial cells, which further brings about the translocation of LPS from the intestine to the systematic circulation (83). Moreover, the decrease of *Akkermansia muciniphila* also contributes to the translocation of microbial byproducts owing to its function in maintaining gut barrier integrity (84). Furthermore, HFD is favorable for the incorporation of LPS into chylomicrons, thus promoting the absorption of LPS in the gut and its transport to the systematic circulation through the lymphatic fluid (85). The afore-mentioned pathways result in elevated LPS levels in circulation.

In systematic circulation, LPS is able to initiate immune responses in adipose tissue and liver. LPS first binds to the LBP, and then forms a complex with CD14 (56). This complex then induces the expression of activator protein 1 and nuclear factor kappa B (NF-kB) by activating TLR4 expressed on macrophage and adipose tissue, which contributes to the secretion of proinflammatory cytokines and chemokines, including TNF-α, IL-6 as well as monocyte chemoattractant protein (MCP)-1 (56, 57). These cytokines can act on adipocytes and stimulate them to secrete more cytokines and chemokines *via* an autocrine and paracrine way (52). Moreover, the overexpression of MCP-1 in adipose tissue has been confirmed to be related to increased macrophage infiltration in rodents (86).

Of note, SCFAs act as another critical linkage between inflammatory responses and the gut microbiota, which exhibit potent anti-inflammation properties, especially butyrate (47, 87–90). Butyrate can protect the gut against inflammation by stimulating IL-18 secretion and promoting regulatory T cells and IL-10-producing T cells differentiation mediated by G protein coupled receptor (GPR) 109a (87, 88). Besides, butyrate is also able to upregulate PPAR-γ and repress NF-kB activation induced by LPS (89, 90), thus exerting its anti-inflammation effects.

In conclusion, gut microbiota alterations in obese subjects result in higher luminal LPS concentration (20), which leads to gut barrier disruption and facilitates bacterial byproducts translocation from the gut to the systematic circulation (83). LPS activates TLR4 on macrophage and adipose tissue, thus contributing to proinflammatory cytokines and chemokines secretion, including TNF-α, IL-6 and MCP-1 (56, 57). However, the increased butyrate in obesity exerts anti-inflammation effects by inhibiting NF-kB activation (89, 90) and inducing anti-inflammatory cytokines production (87, 88). It remains unknown whether the anti-inflammatory effects of SCFAs can partly counteract the chronic inflammation induced by LPS, which warrants further study.

## 4 Long noncoding RNA: Linking the gut microbiota to obesity?

Long noncoding RNA (lncRNA) is identified as a critical regulator in a variety of physiologic and pathologic processes, which has the capacity to control large-scale gene expression programs by interacting with chromatin at numerous different sites, and can modulate the gene expression profile by exerting influence on the stability of mRNA (91–94). The transcription of lncRNA occurs in an independent manner and affects protein-coding genes expression according to the computational analyses (95, 96). In an animal study, the gut microbiota was confirmed to have an intimate association with the constitutive expression of lncRNA in a variety of tissues, such as the gut, muscle, liver and adipose tissues (97). Moreover, the absence of gut microbiota led to the dysregulation of multiple intergenic lncRNAs, indicating that the gut microbiota took part in modulating the epigenetic control of gene expression (97). Furthermore, some researchers have found that fecal microbiota transplantation was able to retain the expression profile of lncRNA in the host (98). Conversely, the lncRNA signatures were conducive to the discrimination of mice with different transplanted microbiota (99).

Many studies have suggested that lncRNA was intimately related to the development of obesity (100–103). The lncRNA dysregulation led to the reduction of leptin, which undermined the afferent signal in the negative feedback loop associated with the maintenance of adipose tissue mass homeostasis, thus resulting in the leptin responsive-obesity (100). In obese mice, the β-cell function and apoptosis regulator, an islet-enriched lncRNA, was significantly downregulated in the islet, which correlated with the dysfunction and apoptosis of β-cell in obesity (101). Besides, several studies have also indicated that the lncRNA played an important role in regulating the inflammatory pathways related to obesity (102). Furthermore, the BAT enriched lncRNA 10 had the capacity to facilitate the browning of white adipose tissue and the activation of BAT, which could fight against obesity to some extent (103).

Taken together, the gut microbiota correlated with the expression profile of lncRNA, and lncRNA participated in the progression of obesity. However, whether the altered gut microbiota in obesity contributes to the disease’s progression partly by regulating lncRNA remains largely unknown, which needs more intensive investigations.

## 5 Potential therapies based on gut microbiota restoration as treatment for obesity

### 5.1 Probiotics

According to WHO, probiotics are defined as the “living microorganisms that provide the host with beneficial effects when administrated in sufficient quantities” (104). Currently, probiotics have been extensively utilized in the prevention and treatment of various diseases, ranging from periodontal diseases to gastrointestinal infections, especially *Lactobacillus* and *Bifidobacterium* species (105, 106). As commensal microorganisms in the human gut, probiotics putatively exert its beneficial effects by competing with pathogenic bacteria, strengthening gut barrier function, and modulating immune responses (107).

Recently, many animal and human studies have also found that probiotics had the capacity to ameliorate metabolic disorders, inflammation conditions, as well as weight gain in obese subjects (108–112). When administrated with *Bifidobacterium pseudocatenulatum* CECT 7765, the inflammatory cascade reaction in obese mice induced by HFD was markedly mitigated compared to the controls (108). And the administration of *Akkermansia muciniphila* has been proved to confer protection against obesity in mice by ameliorating dyslipidemia, insulin resistance, and fat mass development (109). The probiotic VSL#3 encompasses *Lactobacillus* strains and *Bifidobacteria*, which could be used to treat obesity in mouse models by improving insulin resistance, reducing food intake, as well as suppressing body weight gain (110). Similarly, the administration of VSL#3 in human subjects also improved insulin sensitivity and lipid profiles in a randomized controlled trial (111). The probiotic powder *L. plantarum* Dad-13 was able to alter the composition of gut microbiota in a double-blind, placebo-controlled trial, including decreasing Firmicutes abundance and increasing Bacteroidetes abundance, and it also reduced body weight and BMI significantly (112). In another double-blind, randomized trial, the supplementation of the probiotic mix (*Bifidobacterium*, *Lactococcus* as well as *Lactobacillus*) in overweight and obese individuals, increased antioxidant enzyme activity and reduced abdominal adiposity. However, a recent meta-analysis of randomized controlled human studies has reported that the relationship between weight loss and probiotics treatment was insignificant (113). Moreover, which bacterial species and their respective optimal quantity and duration can improve obesity effectively also warrants further investigations.

### 5.2 Prebiotics

Prebiotics refer to indigestible ingredients selectively utilized by host microbiota, which provide beneficial effects for the host mainly *via* alleviating gut dysbiosis (114). Several studies have also shown that prebiotics could improve metabolic disorders, gut dysbiosis and chronic inflammation in obesity (115–118). In obese rats, the administration of prebiotics (oligofructose and inulin) led to secretion of satiety hormones, like PYY and GLP-1, and gut microbiota restoration, including the reduction of Firmicutes abundance and the increment of Bacteroidetes, *Lactobacillus* and *Bifidobacteria* abundance (115). The prebiotic treatment in genetically obese mice was also proved to have a correlation with weight loss, improved glucose tolerance and inflammation condition, increased Bacteroidetes and decreased Firmicutes phylum (116). Similarly, in a double-blind, placebo-controlled trial, the oligofructose-enriched inulin supplementation in overweight or obese children significantly reduced the serum IL-6 level and body weight of such individuals (117). Another randomized, placebo-controlled trial analyzed the components of fecal samples from obese individuals who consumed Inulin-type fructans, and found that *Bifidobacterium* abundance was increased and fecal calprotectin (a marker of gut inflammation) concentration was reduced compared to the controls (118). The afore-mentioned animal and human researches indicate that prebiotics putatively represent a new avenue for obesity treatment, and further studies are still warranted.

### 5.3 Fecal microbiota transplantation

Fecal microbiota transplantation (FMT) is defined as an engraftment of the fecal suspension from healthy donors in patients’ gut tract, which aims to reconstitute the gut microbiota and treat related diseases (119). Unlike probiotics, FMT can provide the recipients with the complete gut microbiota and its byproducts from healthy donors, making it a more effective therapeutic intervention (120). Moreover, the spectacular success of FMT in *Clostridium difficile* infections treatment indicates that FMT is emerging as a potential therapeutic candidate for multiple diseases associated with gut dysbiosis, such as chronic constipation, irritable bowel syndrome and ulcerative colitis (107). An animal study has found that, following the administration of FMT from healthy donors, the metabolic profiles of the obese mice induced by HFD were markedly ameliorated, and that the advantageous effects of exercise and diet could be transferred by FMT (121). Similarly, in a preliminary human study, the peripheral insulin sensitivity of nine obese individuals with metabolic syndrome was significantly improved after transplanted with fecal microbiota from lean donors (13). However, in several recent randomized clinical trials, FMT did not exert significant influences on metabolic profiles and weight loss (122, 123). These controversial reports warrant further investigation. Furthermore, a few studies have reported several FMT-related detrimental events, including vomiting, constipation, diarrhea, and abdominal discomfort (124), raising concerns about FMT’s safety.

In summary, substantial animal and human studies have suggested that probiotics, prebiotics, and FMT could be potential therapeutic interventions for obesity *via* the restoration of the gut microbiota (13, 111, 112, 117, 118, 121). Probiotics ameliorated metabolic disorders, inflammation conditions and weight gain in obese individuals (111, 112). Prebiotics was related to reversing gut dysbiosis and alleviating inflammatory responses (117, 118). FMT had the capacity to alter metabolic profiles and transfer the advantageous effects of exercise and diet from the donor to the recipient (13, 121). Although there are some controversial results concerning the effects of these interventions, they still confer novel insights on the treatment of obesity.

## 6 Conclusion

Currently, obesity has emerged as a serious socioeconomic and public health problem worldwide (2). Owing to its global prevalence and increasing incidence rate, it is urgent to seek for more effective therapeutic interventions (1). An accumulating body of evidence have found that the gut microbiota, as an environmental factor, has been gaining ground as a critical contributor to the etiology of obesity (6–9). The diversity and composition of the gut microbiota have changed significantly in obese subjects compared to the controls. Overall, the gut microbiota in obese subjects exhibits lower diversity, higher Firmicutes abundance, and increased Firmicutes/Bacteroidetes ratio (19, 20). The altered gut microbiota affects the metabolism of luminal contents, such as SCFAs, LPS and bile acids (20, 24, 42, 43). They participate in the pathogenesis of obesity *via* multiple mechanisms, including disrupting energy homeostasis, increasing lipid synthesis and storage, regulating central appetite and feeding behavior, as well as triggering chronic low-grade inflammation. Due to the critical role of the gut microbiota in obesity, some therapeutic interventions based on gut microbiota restoration seem to become potential candidates for obesity treatment, including probiotics, prebiotics and FMT (13, 111, 112, 117, 118, 121).

However, there are still some problems to be solved. Most studies concerning gut microbiota and obesity are mainly carried out in animal models. Although animal models are useful tools to confer insights into underlying disease mechanisms, it remains largely uncertain for them to show human equivalency. Moreover, the paradoxical effects of SCFAs in the development of obesity are another important problem worthy of in-depth investigation. Besides, whether the altered gut microbiota in obesity contributes to the disease’s progression partly by regulating lncRNA remains largely unknown. Furthermore, the safety and efficacy of FMT still need further rigorously evaluations despite its current great potential in the treatment of obesity. Therefore, in the future, more attention should be paid to further exploring the critical bacterial strains affecting obesity and their pathogenic mechanisms. These studies may provide potential clinical value for more precise treatment of obesity, the resolution of current controversial issues, and the formulation of the optimal management based on gut microbiota restoration for obese individuals.

## Author contributions

ZLC collected the literatures and drafted the manuscript. LZ revised the manuscript. LY and HC contributed to the conception and design of the work, and critically revised the manuscript. All authors read and approved the final version of the manuscript.

## Funding

This study was supported by the National Natural Science Foundation of China (No. 82000561 to HC; No. 82270614, No. 81974078, 81570530, 81370550 to LY), Natural Science Foundation of Hubei Province (No. 2019ACA1333 to LY) and the Science foundation of union hospital (No. 2021xhyn005 to HC).

## Acknowledgments

The authors thank Yixin Zhu for the help in polishing the language and grammar.

## Conflict of interest

The authors declare that the research was conducted in the absence of any commercial or financial relationships that could be construed as a potential conflict of interest.

## Publisher’s note

All claims expressed in this article are solely those of the authors and do not necessarily represent those of their affiliated organizations, or those of the publisher, the editors and the reviewers. Any product that may be evaluated in this article, or claim that may be made by its manufacturer, is not guaranteed or endorsed by the publisher.

## Abbreviations

HFD, high-fat diet; LBP, lipopolysaccharide binding protein; SCFAs, short-chain fatty acids; TGR5, Takeda G protein-coupled receptor 5; BAT, brown adipose tissue; FXR, Farnesoid X Receptor; FGF, fibroblast growth factor; FIAF, fasting-induced adipose factor; SREBP1c, sterol regulatory binding protein1c; ChREBP, carbohydrate response element binding protein; LPS, lipopolysaccharide; IL, Interleukin; TNF, tumor necrosis factor; LPL, lipoprotein lipase; PYY, peptide YY; GLP-1, glucogen-like peptide 1; GABA, γ-aminobutyric acid; NF-kB, nuclear factor kappa B; MCP, monocyte chemoattractant protein; GPR, G protein coupled receptor; FMT, Fecal microbiota transplantation; T2DM, type-2 diabetes mellitus; lncRNA, long noncoding RNA.
